# On Dysmenorrhea and Other Uterine Affections in Connexion with Derangement of the Assimilating Functions

**Published:** 1844-10

**Authors:** 


### ART. XIX.

*On Dysmenorrhea and other Uterine Affections in connexion with Derangement of the Assimilating Functions.* By EDWARD RIGBY, M.D. &c.—*London*, 1844. 12mo, pp. 140.

THE liability of puerperal patients to attacks of acute rheumatism has been long observed; and, among the exciting causes of rheumatism, gonorrhea, and the suppression of the catamenia or of leucorrheal discharges, have been frequently noticed. A remarkable connexion between dysmenorrhea and rheumatism has also been discovered, and was particularly pointed out by Dr. Gooch and Dr. Locock, (Cyclopædia of Prac. Medicine—Art. Dysmenorrhea.) The relief afforded by the use of colchicum, and also in a remarkable manner by guaiac, in certain cases of dysmenorrhea, as pointed out by Dr. Dewees and Dr. Gooch, and, as we believe, many years prior to them, by the late Professor Hamilton of Edinburgh University, doubtless led to the discovery of this connexion. The truly valuable researches of Dr. Prout into the morbid products of the urinary secretion in derangement of the digestive organs, and particularly in that form of derangement which is connected with gout and rheumatism, have formed the basis of a further inquiry into the relation between the gouty and rheumatic diathesis and dysmenorrhea. The small work of Dr. Rigby forms a contribution to this inquiry, and a summary of the present extent of our knowledge of this subject.

The first part of the work contains a brief sketch of the results of Dr. Prout's observations on the effect of derangement of the assimilating functions in producing morbid excretions from the skin, mucous membranes, and kidneys. It contains also a short notice of the affections of the different mucous membranes in those states of mal-assimilation which mark the gouty and rheumatic diatheses. This leads to the description of those uterine affections which have been observed in connexion with these diatheses, which forms the second and principal part of the work.

The uterine affections described by Dr. Rigby are of an inflammatory or congestive character. He describes two forms: One of an acute nature, characterized by heat, swelling, redness, and pain of the parts, somewhat sudden in its invasion, and erratic in its movements; occurring mostly in connexion with dysmenorrheal attacks, or at the half-way time between the menstrual periods. Another of a chronic nature, characterized by venous engorgement and swelling; attended mostly with leucorrheal discharge, or subacute inflammation of the cervix uteri; and terminating in induration or ultimately in organic disease. These cases are accompanied also with pain, and sense of fulness and distension in the parts, aggravated by motion or pressure, with frequent discharge of flatus from the vagina, and with well-marked evidence of the gouty or rheumatic diathesis in the urine. A tendency to hemorrhoids, a secretion from the rectum also of gas and an albuminous or mucous fluid similar to the leucorrheal discharge, attends the affection and marks the general congestion of the pelvic viscera.

Of the fibrinous exudations which attend cases of dysmenorrhea, Dr. Rigby confesses himself unable to determine the conditions on which they depend, but expresses his conviction that there is an invariable co-existence of inflammatory action in some neighbouring organ—the kidney, the ovary, or the uterus. Of the immediate cause, we believe, there can be only one opinion, viz. that they depend upon the existence of a certain degree of irritation or inflammatory action in the uterus itself in the production of which, we presume, the state of the ovaries or kidneys can only act secondarily.

We must confess our inability to assent to the statements of Dr. Rigby with regard to the termination of the chronic affections described by him in organic disease, in schirrus of the uterus. We are confident that, on the one hand, such affections may and do continue for an indefinite period without such a termination; and on the other, that cancer invades the uterus without any such previous disease.

We have here to complain, also, of a want of precision in the description of these affections; it is nowhere stated in the work before us what is their precise frequency compared with other affections of a similar kind, and the careless reader might be misled by the general tenor of Dr. Rigby's work to believe that, in his opinion, *all* such affections are of a rheumatic gouty character, and that they are never generated, except under the influence of such a diathesis or habit of body.

In the acute affection Dr. Rigby recommends after free purging with calomel and rhubarb, the application of leeches to the anus or os uteri, emollient and sedative injections and the administration of alkalies. We have seen, in such cases, very decided advantage from free and repeated scarifications of the cervix uteri, a mode of treatment which, from the readiness and certainty with which it can be accomplished after the introduction of a tube or speculum, and the trifling uneasiness it causes, possesses many recommendations, particularly in hospital or dispensary practice.

In the chronic cases characterized by the rheumatic gouty symptoms, Dr. Rigby recommends guiacum and iodine, separately or combined, taraxacum, saline remedies, more particularly the saline mineral waters, lime-water, and muriate of lime; and in the dysmenorrheal paroxysms, the hip warm bath, with pills of camphor, lettuce, and hop, or failing these, opium, as the best means of relieving the sufferings of the patient.

Combined with these remedial means, Dr. Rigby lays down very excellent dietetic rules, and furnishes some valuable practical hints for the management of the different peculiarities of individual cases. The work concludes with brief but very good instructions for the mode in which the urine should be examined, and a description illustrated by plates, of the appearances presented by the products of analysis when placed under the microscope.

The work on the whole, is calculated to be exceedingly useful by directing the profession to rational principles of treatment in the diseases referred to. Although it does not pretend to the development of any original views regarding those diseases, or any new discoveries regarding their mode of treatment, it is well adapted to impress upon the attention of practitioners, the importance of constitutional treatment, in a class of diseases, which are, in an especial manner, dependent upon constitutional causes. Something has been done of late years, and much yet remains to be done, in forming correct notions of the pathology of certain diseases, the most prevalent diseases of civilized life, by an examination into the changes produced on the products of excretion, by disorders in the functions of assimilation. To Dr. Prout, the profession is under deep and lasting obligations for his labours in this field of inquiry, and to all who prosecute investigations in the same direction, we are bound to offer our meed of praise, and hold out every encouragement to proceed.

